# New Lignans from the Leaves and Stems of *Kadsura philippinensis*

**DOI:** 10.3390/molecules18066573

**Published:** 2013-06-04

**Authors:** Yu-Chi Lin, Yuan-Bin Cheng, Chia-Ching Liaw, I-Wen Lo, Yao-Haur Kuo, Michael Y. Chiang, Chang-Hung Chou, Ya-Ching Shen

**Affiliations:** 1 School of Pharmacy, College of Medicine, National Taiwan University, 1, Sec. 1, Jen-Ai Rd., Taipei 100, Taiwan; E-Mail: z10108042@email.ncku.edu.tw (Y.-C.L.); jmb@kmu.edu.tw (Y.-B.C.); biogodas@hotmail.com (C.-C.L.); d96423006@ntu.edu.tw (I.-W.L.); 2 Department of Life Sciences, National Cheng Kung University, No. 1 University Road, Tainan 701, Taiwan; 3 Graduate Institute of Natural Products, School of Pharmacy, Kaohsiung Medical University, Shih-Chuan 1st Road, Kaohsiung 807, Taiwan; 4 National Research Institute of Chinese Medicine, Taipei 112, Taiwan; E-Mail: kuoyh@nricm.edu.tw; 5 Department of Chemistry, National Sun Yat-sen University, 70, Lien-hai Road, Kaohsiung 804, Taiwan; E-Mail: michael@mail.nsysu.edu.tw; 6 Graduate Institute of Ecology and Evolutionary Biology, China Medical University, Taichung 40402, Taiwan; E-Mail: choumasa@mail.cmu.edu.tw

**Keywords:** *Kadsura philippinensis*, taiwankadsurins, lignans

## Abstract

Three novel C19 homolignans, taiwankadsurins D (**1**), E (**2**) and F (**4**), and two new C18 lignans kadsuphilins N (**3**) and O (**5**) were isolated from the aerial parts of Taiwanese medicinal plant *Kadsura philippinensis*. The structures of compounds **1**–**5** were determined by spectroscopic analyses, especially 2D NMR techniques. The structure of compound **5** was further confirmed by X-ray crystallographic analysis. Compounds **1** and **2** have a 3,4-{1'-[(Z)-2''-methoxy-2''-oxoethylidene]}-pentano(2,3-dihydrobenzo[b]furano)-3-(2'''-methoxycarbonyl-2'''-hydroxy-2''',3'-epoxide) skeleton.

## 1. Introduction

*Kadsura* belongs to the family Schisandraceae and it is only distributed in eastern and southern Asia [1]. Species of *Kadsura* were used in Chinese folk medicine for the treatment of cold, rheumatoid arthritis and gastroenteritis and as an anodyne to relieve pain [2]. The major constituents of *Kadsura* plants were reported to be bioactive lignans, which possess antitumor, antiviral and anti-hepatitic activities [3,4,5,6,7,8]. *K*. *philippinensis* Elm. is an evergreen vine, mainly distributed at low altitude onremote islands of Taiwan such as Green Island [9]. Our previous phytochemical studies on the EtOAc extracts of *K. philippinensis* resulted in the isolation of two novel triterpene dilactones and many lignans [10,11,12,13,14,15,16,17]. In this paper, we report the isolation and structure elucidation of three new C19 homolignans, named taiwankadsurins D-F, and two new C18 lignans, designated kadsuphilins N and O.

## 2. Results and Discussion

**Figure 1:**
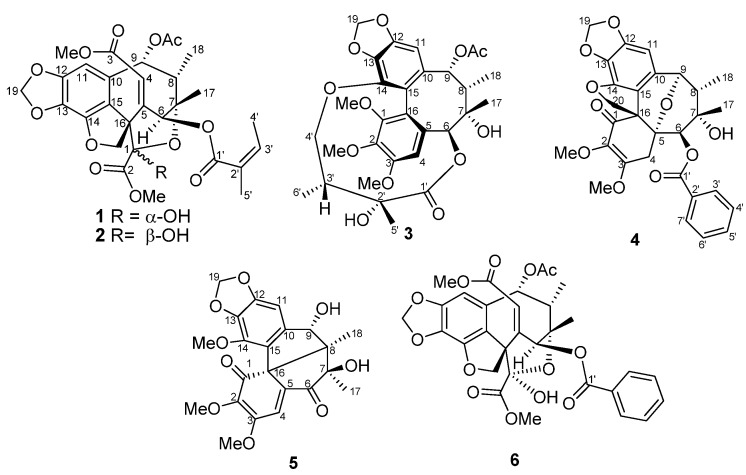
Chemical structures of compounds **1**–**6**.

The leaves and stems of *K. philippinensis* were extracted with mixture of CH_2_Cl_2_ and acetone, then suspended in H_2_O and extracted with EtOAc. The EtOAc-soluble part was subjected to extensive chromatography including flash column, normal and reversed-phase HPLC, furnishing compounds **1**–**5** (Figure 1).

**Figure 2:**
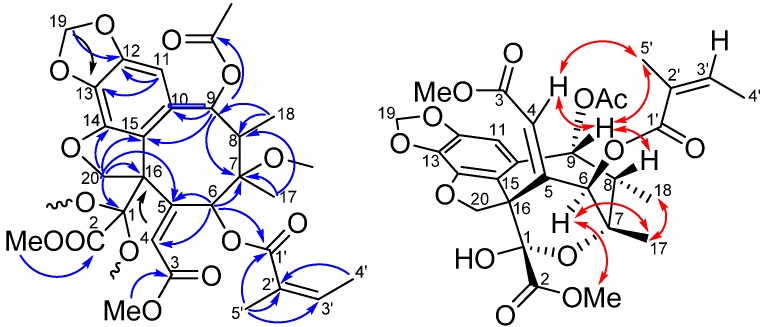
Selected HMBC (arrow) and NOESY (double headed arrow) correlations of **1**.

*Taiwankadsurin D* (**1**), (${\left[\alpha \right]}_{D}^{25}$ +57°, CH_2_Cl_2_) had a molecular formula C_29_H_32_O_13_, as derived from its HREIMS at *m/z* 611.1735 ([M+Na]^+^, calcd 611.1741) indicating 14 degrees of unsaturation. The UV absorption (273, 225 nm) and IR bands (1,731, 1,721 and 1,628 cm^−1^) indicated a benzyl and α,β, unsaturated ester functionalities. The ^1^H-NMR of **1** exhibited two methoxyl singlets (δ 3.93, 3.59), an acetyl singlet (δ 2.13), two methyl singlets (δ 1.31, 1.99), two methyl doublets (δ 1.36, *J* = 6.9 Hz; δ 2.05, *J* = 7.2 Hz), two oxymethylene protons (δ 5.00, 4.53, each d, *J* = 10.2 Hz) and two dioxymethylene protons (δ 5.97, 5.98, each s-like). According to ^13^C-NMR and DEPT spectra, compound **1** had total 29 signals including seven methyl, two methylene, six methine and fourteen quaternary carbons. Moreover, ^1^H-NMR spectroscopic data of **1** showed characteristic signals of H-4 (δ 5.99), H-6 (δ 6.28) and H-9 (δ 6.55), and ^13^C-NMR data of C-1 (δ 97.5 s), C-2 (δ 171.0 s) and C-3 (δ 165.4 s) similar to those of taiwankadsurin A (**6**), suggesting that compound **1** is an analogue of the latter [10]. However, a benzoyl group in **6** was missing and replaced with an angeloyl group at C-6 in **1**. Further HMBC correlations (Figure 2) of H-11/C-12, C-13, C-15 and H-20/C-14, C-15, C-16, confirmed that compound **1** possessed a dihydrobenzofuran system. The ethylidene-octane ring was also deduced from the HMBC correlations of H-9/C-7,C-10,C-11,C-15; Me-18/C-7,C-8,C-9; Me-17/C-6,C-7,C-8 and H-6/C-4,C-5, C-7, C-8. The acetyl and angeloyl groups attaching at C-9 and C-6 respectively, were resulted from the HMBC correlations of H-9 (δ 6.55) with the acetyl carbonyl, and H-6 (δ 6.28) with the angeloyl carbonyl. Furthermore, methoxyl groups (δ_Η_ 3.93, δ_Η_ 3.59) attaching at carbonyls C-2 (δ_C_ 171.0) and C-3 (δ_C_ 165.4) were deduced from their mutual HMBC correlations.

It was noted that the dioxygenated tertiary carbon C-1 connected to C-7 through an ether bridge to account for the last degree of unsaturation. The relative configuration of **1** was determined by the NOESY experiment and by comparing the NMR data of **1** with those of taiwankadsurin A (Figure 2). Assuming that H-9 was β-oriented due to quite similar NMR spectra of **1** and taiwankadsurin A [10], thus, cross peaks between H-4, H-9 and Me-5', and correlation between H-9 and H-8, rather than Me-18 suggested that H-8 and 6-*O*-angeloyl group should be positioned on the β−face of the molecule. On the other hand, correlation between Me-18(eq) and Me-17(eq) accounted for the α−disposition of the ether ring between C-1 and C-7. In addition, NOESY correlation between H-6 and the methoxyl protons at C-2 indicated that H-6 and the hydroxyl group attached at C-1 are α−oriented. On the basis of above findings, the relative configuration of **1** was assigned as 1R*, 6S*, 7S*, 8S*, 9R*, 16S*.

*Taiwankadsurin E* (**2**) is an isomer of **1** as inferred from the identical molecular weight in HRMS, similar UV and IR absorptions and NMR data. The ^1^H-NMR spectrum (Table 1) of **2** had the same characteristic peaks with **1** except that H-6 was downfield shifted to δ_Η_ 6.91, while the methoxyl protons at C-2 was upfield shifted to δ_Η_ 3.61. Detail analysis of HMBC correlations of **2** revealed that the locations of angeloyl, acetyl and methoxyl groups were the same as **1**. The configuration of **2** was established from NOESY experiment, in which most of the cross peaks were identical to those of **1**. However, the correlation between H-6 and the methoxy at C-2 was missing in **2**. Therefore, the structure of **2** was established, being an 1-epimer of **1**.

**Table 1:** ^1^H-NMR data (CDCl_3_) of compounds **1**–**5** *^a,b^*.

Position	1 *^a^*	2 *^b^*	3 *^a^*	4 *^b^*	5 *^a^*
4	5.99, brs	6.06, d (2.4)	6.84, s	3.08, d (18.4 )	7.34, s
				3.17, d (18.4 )	
6	6.28, d (2.7)	6.69, d (2.4)	5.76, s	5.42, s	
8	2.23, m	2.23, m	1.97, m	2.00, m	
9	6.55, d (2.7)	6.63, d (2.8)	5.48, s	4.82, brs	4.87, d (12.9)
11	6.45, s	6.44, s	6.47, s	6.28, s	6.71, s
17	1.31, s	1.34, s	1.37, s	0.97, s	1.31, s
18	1.04, d (6.9)	1.02, d (6.8)	1.30, d (6.9)	1.36, d (7.6)	0.98, s
19	5.97, s	5.93, s	5.94, s	5.83, s	5.90, d (1.5)
	5.98, s	5.94, s	6.03, s	5.98, s	5.91, d (1.5)
20	4.53, d (10.2)	4.59, d (10.0)		4.30, d (9.6)	
	5.00, d (10.2)	4.98, d (10.0)		4.43, d (9.6)	
OMe-1			3.46, s		
OMe-2	3.93, s	3.57, s	3.84, s	3.66, s	3.77, s
OMe-3	3.59, s	3.58, s	3.92, s	4.07, s	4.11, s
OMe-14					3.81, s
OAc	2.13, s	2.13, s	1.49, s		
1'					
2'					
3'	6.28, overlap	6.23, q (7.2)	1.92, m	7.32, m	
4'	2.05, d (7.2)	2.06, d (7.2)	3.63, dd (5.0, 8.0)	7.35, m	
			4.16, dd (5.0, 8.0)		
5'	1.99, s	2.00, s	1.23, s	7.55, d (7.2)	
6'			0.96,d (7.2)	7.35, m	
7'				7.32, m	
OH-9					4.28, d (12.9)

*^a^* recorded at 300 MHz. *^b^* recorded at 400 MHz.

**Figure 3:**
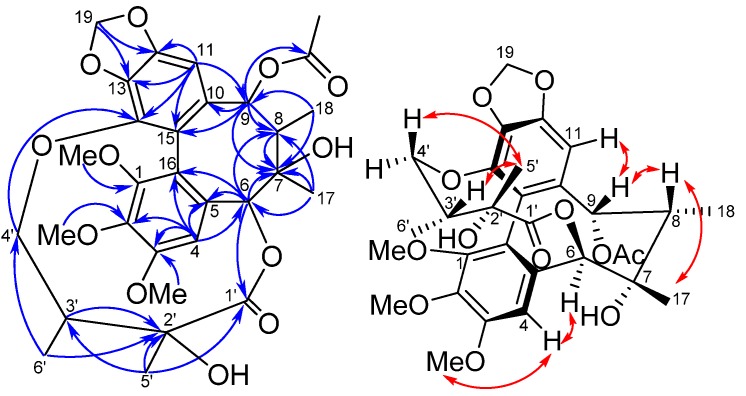
Selected HMBC (arrow) and NOESY (double headed arrow) correlations of **3**.

*Kadsuphilin N* (**3**), (${\left[\alpha \right]}_{D}^{26}$ −2.4, CH_2_Cl_2_), had a molecular formula of C_30_H_36_O_12_ as deduced from a *pseudo*-molecular ion [M+Na]^+^ at *m/z* 611.2107 in the HRESIMS. The UV absorption bands at 212, 259 and 292 nm suggested that **1** possessed a biphenyl chromophore. The IR absorption indicated the presence of hydroxyl (3,479 cm^−1^) and carbonyl (1,738 cm^−1^) groups. The ^13^C-NMR spectroscopic data and DEPT analysis revealed that compound **3** contains 30 carbons, including ten quaternary sp^2^ carbons (δ_C_ 121.2, 121.7, 130.8, 132.8, 137.5, 139.0, 141.8, 148.6, 151.5 and 152.3), two ester carbons (δ_C_ 168.7 and 172.4), two quaternary sp^3^ oxygen-bearing carbons (δ_C_ 73.8 and 76.6), two oxygen-bearing methylene carbons (δ_C_ 72.4 and 101.5), two sp^2^ methine carbons (δ_C_ 102.5 and 111.0), two sp^3^ methine carbons (δ_C_ 42.4 and 44.1), two oxygen-bearing sp^3^ methine carbons (δ_C_ 83.9 and 86.6), and eight methyl groups (δ_C_ 12.8, 17.8, 20.3, 21.4, 28.4, 56.2, 60.5 and 60.7). The HMBC correlations of H-11/C-9, C-10, C-12, C-13, C-15; H-9/C-10, C-15; H-4/C-2, C-3, C-5, C-6, C-16; H-6/C-5, C-16; Me-17/C-6, C-7, C-8; Me-18/C-7, C-8, C-9 implied that compound **3** indeed possessed a schizandrin type dibenzocyclooctadiene system [16]. Moreover, HMBC correlations of H_2_-19/C-12, C-13; OMe-1/C-1; OMe-2/C-2; OMe-3/C-3 and H-9/ acetyl carbonyl assigned the methylenedioxy group and three methoxyl groups attached to the aromatic ring and an acetyl group at C-9. In addition, the ester linkage could be proved by correlations of H-6/C-1'; Me-5'/C-1', C-2', C-3'; Me-6'/C-2', C-3', C-4' and H-4'/C-14. From the above interpretation, the structure of **3** could be established as 9-acetylgomisin D. The configuration was determined by CD spectrum and NOESY experiment. The strong positive Cotton effect at 229 nm and the negative Cotton effect at 245 nm assigned the *S*-configuration of the biphenyl system [18]. The NOESY correlations of H-9/H-8, H-11, H-8/Me-17 and H-6/H-4, Me-17(eq) revealed that the cyclooctadiene ring had a twist-boat-chair form and H-8, H-9 and Me-17 were β-oriented while H-6 and Me-18 were α-configuration (Figure 3). The correlations of H-3'/Me-5' and the NMR data were in good agreement with the configuration of ester linkage that was also present in gomisin D [19].

**Figure 4:**
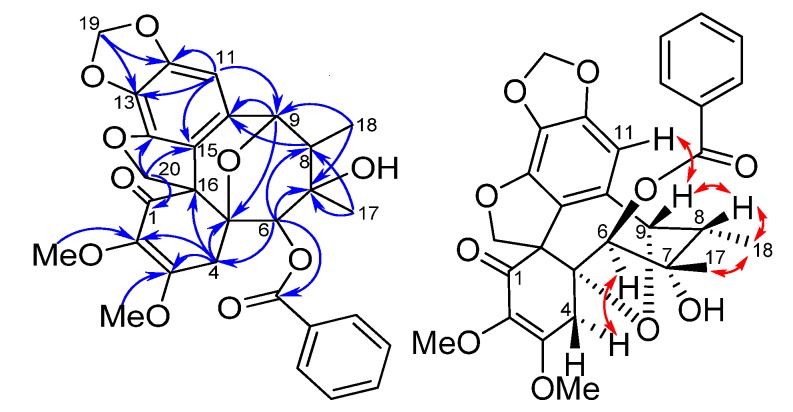
Selected HMBC (arrow) and NOESY (double headed arrow) correlations of **4**.

*Taiwankadsurin F* (**4**) was isolated as a pale yellow amorphous solid. The molecular formula C_29_H_28_O_10_ was deduced from a *pseudo*-molecular ion at *m/z* 559.1580 [M+Na]^+^ in HRESIMS. The UV spectrum showed absorptions at *λ*_max_ 255, 220 nm and IR bands at *ν*_max_ 3,510, 1,735, 1,725 cm^−1^ suggested that compound **4** contained phenyl, benzoyl, α,β-unsaturated ketone and hydroxyl functionalities. The ^1^H and ^13^C-NMR spectroscopic data (Table 1 , Table 2) revealed that **4** possessed a substituted cyclohex-2-enone moiety and a spirodihydrobenzofuran ring in a homolignan skeleton similar to kadsuphilol G [14]. The difference could be the angeloyloxy side chain, which was substituted with a benzoyloxy group. This scaffold was supported from HMBC correlations of H-19/ C-12, C-13; H-11/ C-9, C-12, C-13, C-15; Me-18/C-7, C-8, C-9; Me-17/C-6, C-7, C-8; H-4/C-2, C-3, C-5, C-16 and H-20/C-1, C-14, C-15. Moreover, the key HMBC correlations of H-6/ benzoyl carbonyl (δ_C_ 165.4) and H-9/ C-5 assigned the benzoyl group at C-6. It was found that an ether linkage appeared between C-5 and C-9 due to calculation of double bond equivalence. The relative configuration of **4** was determined by comparing the coupling constants of **4** with those of kadsuphilol G and NOESY experiments. Thus a twist-boat-chair configuration was elucidated on the basis of CD observation, in which a positive Cotton effect was found at 216 nm and a negative one at 249 nm. The NOESY correlations of H-11/H-8 /H-9 and H-8/ Me-17 suggested that H-8, H-9 Me-17 were all in β-face while Me-18 was α-oriented (Figure 4). Because compound **4** had a TBC-*S* configuration, the oxygen bridge could be assigned as α-disposed. On the basis of the above interpretation, the structure of compound **4** was established and the name taiwankadsurin F was given.

**Table 2:** ^13^C-NMR data (CDCl_3_) of compounds **1**–**5** *^a,b^*.

Position	1 *^a^*	2 *^b^*	3 *^a^*	4 *^b^*	5 *^a^*
1	97.5, C	97.6, C	151.5,C	193.0, C	196.3, C
2	171.0, C	170.1, C	141.8,C	132.5, C	140.6, C
3	165.4, C	165.5, C	152.3, C	157.4, C	159.0, C
4	117.2, CH	118.3, CH	111.0, CH	40.7, CH_2_	123.4, CH
5	150.5, C	149.8, C	130.8, C	77.6, C	143.7, C
6	72.5, CH	72.5, CH	86.6 , CH	77.3, CH	200.7, C
7	79.2, C	78.4, C	73.8, C	72.5, C	80.7, C
8	45.4, CH	45.5, CH	44.1, CH	43.7, CH	60.4, C
9	70.3, CH	70.2, CH	83.9, CH	77.3, CH	75.7, CH
10	127.9, C	127.8, C	132.8,C	127.9, C	144.5, C
11	98.7, CH	99.9, CH	102.5, CH	95.9, CH	100.4, CH
12	150.4, C	149.8, C	148.6, C	151.3, C	151.2, C
13	129.1, C	128.9, C	137.5, C	129.5, C	136.4, C
14	144.9, C	142.6, C	139.0, C	140.9, C	139.5, C
15	118.0, C	120.5, C	121.2, C	121.3, C	125.7, C
16	57.0, C	58.6, C	121.7, C	56.9, C	69.5, C
17	28.2, CH_3_	28.5, CH_3_	28.4, CH_3_	23.1, CH_3_	19.9, CH_3_
18	8.9, CH_3_	8.5, CH_3_	17.8, CH_3_	15.3, CH_3_	15.1, CH_3_
19	101.8, CH_2_	101.5, CH_2_	101.5, CH_2_	101.3, CH_2_	101.7, CH_2_
20	80.4, CH_2_	78.5, CH_2_		78.2, CH_2_	
OMe-1			60.5, CH_3_		
OMe-2	53.6, CH_3_	54.0, CH_3_	60.7, CH_3_	60.7, CH_3_	60.2, CH_3_
OMe-3	51.8, CH_3_	51.7, CH_3_	56.2, CH_3_	58.9, CH_3_	58.3, CH_3_
OMe-14					59.4, CH_3_
OAc	168.9, C	168.9, C	168.7, C		
	21.0, CH_3_	20.3, CH_3_	20.3, CH_3_		
1'	166.0, C	166.1, C	172.4, C	165.4, C	
2'	126.3, C	126.5, C	76.6, C	129.5, C	
3'	142.3, CH	141.7, CH	42.4, CH	128.3, CH	
4'	16.0, CH_3_	15.9, CH_3_	72.4, CH_2_	129.7, CH	
5'	20.4, CH_3_	21.0, CH_3_	21.4, CH_3_	133.9, CH	
6'			12.8, CH_3_	129.7, CH	
7'				128.3, CH	

*^a^* recorded at 75 MHz. *^b^* recorded at 100 MHz.

**Figure 5:**
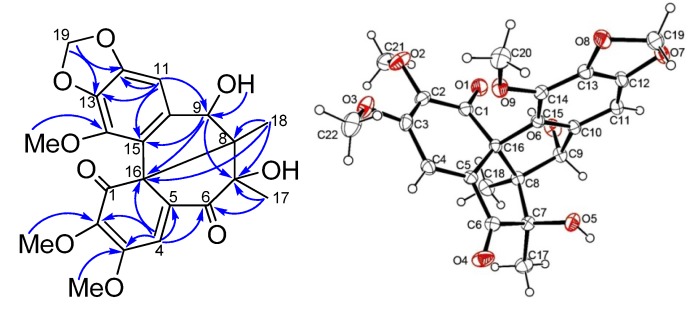
Selected HMBC correlations and X-ray perspective drawing of **5**.

*Kadsuphilin O* (**5**) was obtained as pale yellow crystals, with molecular formula C_22_H_22_O_9_ as determined by HRESIMS (12 degrees of unsaturation). IR absorption bands at 3,420, 1,716 and 1,620 cm^−1^ indicated the presence of hydroxyl, carbonyl and aromatic moieties. The ^1^H-NMR data (Table 1) and HMQC spectrum showed characteristic signals for two aromatic (δ_H_ 6.71, 7.34), one methoxyene-dioxy (δ_H_ 5.90, 5.91 as an AB quartet), one oxygen-bearing methine (δ_H_ 4.87), two *tert*-methyl (δ_H_ 0.98, 1.31) and three methoxyl (δ_H_ 3.77, 3.81 and 4.11) protons. A methine doublet at δ_H_ 4.28 (*J* = 12.9 Hz) revealed the presence of a hydroxy due to no correlation was found in HMQC. ^13^C-NMR data and DEPT spectra revealed that compound **5** contained five pairs of double bonds (δ_C_ 100.4, 123.4, 125.7, 136.4, 139.5, 140.6, 143.7, 144.5, 151.2, 159.0), two ketone carbonyl carbons (δ_C_ 196.3 and 200.7) and three quaternary carbons (δ_C_ 60.4, 69.5, 80.7), one oxygenated methine carbon (δ_C_ 75.7), a methylenedioxy carbon (δ_C_ 101.7), two methyl carbons (δ_C_ 15.1 and 19.9) and three methoxyl carbons (δ_C_ 58.3, 59.4, 60.2). Thus compound **5** possessed five ring systems after deduction of seven double bonds. In the HMBC spectrum, correlations of H-11/C-9, C-12, C-13, C-14, C-15; H-19/C-12, C-13; H-4/C-2, C-3, C-5, C-6, C-16; Me-17/C-6, C-7, C-8; Me-18/C-7, C-8; H-9/C-7, C-15 and C-9-OH/C-9 suggested a dibenzocyclo-octadiene framework with a ketone substituted at the C-6 position. Furthermore, the linkage between C-8 and C-16 was deduced by the correlations of H-9 and Me-18 with C-16, and the remaining ketone group could be assigned to the C-1 position. This finding was further confirmed by comparing the NMR data with those of heteroclitin G [20]. The relative configuration of **5** was determined by NOESY correlation and CD. The CD spectrum of **5** was similar to that of kadsutherin C [21]. The negative Cotton effect at 240 nm and the positive Cotton effect at 218 nm accounted for *S*-configuration for the biphenyl skeleton. Assuming that the H-9 of **5** was in a β-orientation similar to heteroclitin G, the NOESY correlations of HO-9/Me-18 and Me-18/Me-17 indicated that they were on the α-face and OH-7 was β-oriented. Therefore, the configuration of the bipentacyclic ring was established. The structure of **5** was finally confirmed by a single-crystal X-ray diffraction analysis, from which a perspective drawing of **5** is provided in Figure 5.

## 3. Experimental

### 3.1. General

Melting points were measured on a Büchi melting point B-540 apparatus and are uncorrected. Optical rotations were recorded on a JASCO DIP-1000 polarimeter. IR and UV spectra were measured on HORIBA FT-720 and U-3210 spectrophotometers, respectively. The ^1^H- and ^13^C-NMR, COSY, HMQC, HMBC, and NOESY spectra were recorded respectively on a Bruker FT-300 spectrometer (300 MHz for ^1^H and 75 MHz for ^13^C) or on a Bruker AVANCE 400 (400 MHz for ^1^H and 100 MHz for ^13^C) using TMS as an internal standard. The chemical shifts were given in δ values (ppm) and coupling constants in Hz. Low-resolution FABMS were recorded on a VG Quattro 5022 mass spectrometer, and HREIMS were measured on a JEOL JMS-SX 102 spectrometer. Silica gel 60 (Merck) was used for column chromatography (CC), and precoated silica gel plates (Merck, Kieselgel 60 F-254, 1 mm) were used for preparative TLC.

### 3.2. Plant Material

The leaves and stems of *K. philippinensis* were collected at Green Island, Taiwan, in November, 2002. A voucher sample (specimen code: TP 93-2) was deposited at the School of Pharmacy, College of Medicine, National Taiwan University, Taipei, Taiwan.

### 3.3. Extraction and Isolation

*K. philippinensis* was extracted with mixture of CH_2_Cl_2_ and acetone and partitioned between EtOAc and H_2_O (1:1). The EtOAc-soluble part was subjected to Si gel column chromatography (*n*-hexane/EtOAc, 1:0 to 0:1), and after monitoring by ^1^H-NMR, the middle fraction (fr. 21) was further eluted on LH-20 (MeOH) to give five subfractions (fr.21-1~5). Fr.21-5 was chromatographed on a flash column (Si gel, *n*-hexane/EtOAc, 15:1-0:1) and further separated by normal phase HPLC (*n*-hexane/CH_2_Cl_2_/MeOH, 35:65:1) to furnish taiwankadsurins D (**1**, 13 mg) and E (**2**, 2 mg). Kadsuphilin N (**3**, 14 mg) was isolated from fr.21-2, which was chromatographed on a flash column (*n*-hexane/ acetone/EtOAc, 15:1:1 to 1:1:1) and further purified with normal phase HPLC (*n*-hexane/CH_2_Cl_2_/ MeOH, 30:70:1) and reverse phase HPLC (MeOH/H_2_O, 65:35) alternatively. Fraction fr.21-4 was separated on a Si gel column (*n*-hexane/EtOAc, 25:1 to 0:1) and a reverse phase HPLC (MeOH/H_2_O, 65:35) column to yield taiwankadsurin F (**4**, 4 mg) and kadsuphilin O (**5**, 7 mg).

### 3.4. Spectroscopic Data

*Taiwankadsurin D* (**1**). ${\left[\alpha \right]}_{D}^{26}$ +57 ° (*c* 0.5, CH_2_Cl_2_); UV λ_max_ (MeOH) 225, 273 nm; CD (MeOH, *c* = 0.2) nm (ε) 222 (−1.30), 254 (+1.27); IR (neat) ν_max_ 3,450, 2,938, 1,731, 1,721, 1,628 cm^−1^; ^1^H-NMR and ^13^C-NMR (CDCl_3_, 300/75 MHz) see Table 1 , Table 2, respectively; HRESIMS *m/z* 611.1735 (calcd for C_29_H_32_O_13_Na, 611.1741).

*Taiwankadsurin E* (**2**). ${\left[\alpha \right]}_{D}^{26}$ −11° (*c* 0.2, CH_2_Cl_2_); UV λ_max_ (MeOH) 233, 276 nm; CD (MeOH, *c* = 0.2) nm (ε) 228 (−0.78), 247 (+0.27); IR (neat) ν_max_ 3,457, 1,728, 1,717 cm^−1^; ^1^H-NMR and ^13^C-NMR (CDCl_3_, 400/100 MHz) see Table 1 , Table 2, respectively; HRESIMS *m/z* 611.1737 (calcd for C_29_H_32_O_13_Na, 611.1741).

*Kadsuphilin N* (**3**). ${\left[\alpha \right]}_{D}^{25}$ −2.4° (*c* 1.3, CH_2_Cl_2_); UV λ_max_ (MeOH) 212, 259, 292 nm; CD (MeOH, *c* = 0.16) nm (ε) 229 (+33.56), 245 (−2.97), 293 (−5.54); IR (neat) ν_max_ 3,479, 1,738, 1,624, 1,594 cm^−1^; ^1^H-NMR and ^13^C-NMR (CDCl_3_, 300/75 MHz) see Table 1 , Table 2, respectively; HRESIMS *m/z* 611.2107 (calcd for C_30_H_36_O_12_Na, 611.2104).

*TaiwankadsurinF* (**4**). ${\left[\alpha \right]}_{D}^{25}$ −13.2° (*c* 0.6, CH_2_Cl_2_); UV λ_max_ (MeOH) 220, 255 nm; CD (MeOH, *c* = 0.3) nm (ε) 216 (+17.34), 249 (−8.77), 290 (−1.53); IR (neat) ν_max_ 3,510, 1,735, 1,725, 1,660, 1,580 cm^−1^; ^1^H-NMR and ^13^C-NMR (CDCl_3_, 400/100 MHz), see Table 1 , Table 2, respectively; HRESIMS *m/z* 559.1573 (calcd for C_29_H_28_O_10_Na, 559.1580).

*Kadsuphilin O* (**5**). ${\left[\alpha \right]}_{D}^{25}$ −8.0° (*c* 0.6, CH_2_Cl_2_); MP 167 °C; UV λ_max_ (MeOH) 215, 246, 283 nm; CD (MeOH, *c* = 0.22) nm (ε) 218 (+7.66), 240 (−28.11), 282 (−6.75); IR (neat) ν_max_ 3,420, 1,716, 1,620 cm^−1^; ^1^H-NMR (CDCl_3_, 300 MHz) and ^13^C-NMR (CDCl_3_, 75 MHz), see Table 1 and Table 2, respectively; HRESIMS *m/z* 453.1158 (calcd for C_22_H_22_O_9_Na, 453.1161). Crystal data: C_22_H_22_O_9_, *M* = 430.40, trigonal system, space group *P*2_1_, *a* = 10.706(2), *b* = 8.218(2), *c* = 10.9345(9) Å, *V* = 960.1(3) Å^3^, *Z* = 2, *d* = 1.489 Mg/cm^3^. A crystal of dimensions 0.60 × 0.60 × 0.20 mm was used for measurements on a RIGAKU AFC7S diffractometer with a graphite monochromator (ω-2θscans, 2θ_max_= 52.0°), Mo Kα radiation. The total number of independent reflections measured was 2,134, of which 2026 were observed (|*F*|^2^ ≥ 2*σ*|*F*|^2^). The crystal structure was solved by the direct method SHELX-86 [22] and expanded using difference Fourier techniques, refined by the program SHEXTL-97 [23] and full-matrix least-squares calculations. Final indices: *R_f_* = 0.030, *R*_w_ = 0.0784, *w* = 1/[*σ^2^* (*F_o_*^2^) + (0.070*P*)^2^ + 0.1457*P*], where *P* = (*F_o_*^2^ + 2 *F_c_*^2^)/3). Copies of the deposited crystal data (CCDC 829589) can be obtained, free of charge, from the Cambridge Crystallographic Data Centre, 12 Union Road, Cambridge CB2 1EZ, UK; fax: +44 (0) 1223 336033 or E-Mail: deposit@ccdc.cam.ac.uk.

## 4. Conclusions

Phytochemical investigation of the aerial part of Taiwanese *Kadsura philippinensis* has resulted in isolation of five new lignans **1**–**5**, including three novel C19 homolignans, designated taiwankadsurins D, E and F*.* Their structures have been established by spectroscopic analyses, especially 2D NMR techniques. In addition, the structure of compound **5** was further confirmed by X-ray crystallographic analysis.
